# Urinary Schistosomiasis in Urban and Semi-Urban Communities in South-Eastern Nigeria

**Published:** 2013

**Authors:** Ogochukwu Caroline OKEKE, Patience Obiageli UBACHUKWU

**Affiliations:** Parasitology Unit, Dept. of Zoology and Environmental Biology, University of Nigeria, Nsukka, Nigeria

**Keywords:** Prevalence, *S. haematobium*, *Bulinus globosus*, Micro-haematuria, Nigeria

## Abstract

**Background:**

In view of the massive rural-to-urban migration in Nigeria, investigations on transmission of urinary schistosomiasis were carried out in urban and semi-urban communities in Nike Lake area of Enugu State, Nigeria.

**Methods:**

Urine samples of school children were tested for micro-haematuria using reagent strips followed by microscopic examination for *Schistosoma haematobium* eggs. Water contact sites were also identified and sampled for snails.

**Results:**

The overall prevalence of *S. haematobium* eggs in school children was 4.64%. The mean intensity of infection was 1.14 ± 0.41 eggs/10ml urine. Males had insignificantly higher prevalence and intensity of *S. haematobium* infection than females. The youngest age group (4-7 years) had no infection. The prevalence of micro-haematuria (6.2%) was higher than that of microscopy, and this correlated positively with prevalence (r = 0.65, *P* < 0.01) and intensity (r = 0.50, *P* < 0.01) of the infection. Potential intermediate host of human shistosome collected were: *Bulinus globosus, B. senegalensis* and *Biomphalaria pfeifferi*. However, only *B. globosus* shed cercariae of *S. haematobium*, with a snail infection rate of 0.73%. Transmission was in the dry season coinciding with the drying of wells.

**Conclusion:**

The results revealed that urinary schistosomiasis is prevalent, and that *B. globosus* and not *B. truncatus* as previously reported is the main intermediate host of urinary schistosomiasis in this part of Enugu State.

## Introduction

Schistosomiasis is one of the prevalent human parasitic diseases in the world, second only to malaria in terms of socio-economic and public health importance. It is endemic in 76 countries in the tropics and subtropics, and is estimated that there are in excess of 200million people infected, with children being at particular risk (1). In sub-Saharan Africa, Nigeria has the largest number of disability-adjusted life years (DALY) lost to schistosomiasis (2). The current control strategy recommended by the WHO is to control morbidity due to schistosomiasis by mass drug administration (MDA) with praziquantel, targeting mainly school-age children and adult at high risk of infection (3). To ensure success of MDA programme in Nigeria, there is need to update information regarding the prevalence and distribution of urinary schistosomiasis in the country (4).

In Enugu State Nigeria, records of urinary schistosomiasis are mostly from rural areas. A prevalence rate of 79% was reported for *S. haematobium* infection in Amangunze village in Enugu State, Nigeria in 1989 by (5), with *Bulinus truncatus* as the intermediate host. In 1994 and 1995, the prevalence of the infection in Amangunze ranged from 46.6 to 76.0% (6). In Obollo-Eke, a rural community in the State, the prevalence of *S. haematobium* infection was 17.5% while the prevalence of haematuria was 15.6%. The snail host species was not highlighted in the study (7).

In view of the current massive rural-to-urban migration in Nigeria, our study reports the status of urinary schistosomiasis in urban and semi-urban parts of Enugu State, Nigeria.

## Material and Methods

### Study area

Nike Lake area is Nkwo Nike the headquarters of Enugu East Local government area (L.G.A) of Enugu State, Nigeria. Nkwo Nike lies between longitudes 7^0^ 36’ and 7^0^ 41'E and latitudes 6^0^ 31’ and 6^0^ 36'N and is made up of two urban (Amorji and Trans-Ekulu) and four semi-urban communities (Ibagwa, Mbuluowehe, Edem and Amokpo). Nkwo Nike has tropical rain forest vegetation. The climate is humid and the humidity is highest between March and November. The area has two weather conditions of rainy and dry seasons. The average rainfall during rainy season is around 400 mm. One characteristic geographical feature of Nkwo Nike is the presence of a Lake called Nike Lake situated in Amorji. Close to the Lake is the popular Nike Lake resort. From the six communities, three communities were randomly chosen, Amorji, Ibagwa and Amopko. Sources of water in these communities are water from tankers, who get water from bore-hole, and wells but these wells dry up in dry season. Petty trading, civil service and farming are the main occupation of those living in these communities. There is no treatment history and knowledge of urinary schistosomiasis in Nkwo Nike.

### Study population and subject selection

The study is a cross-sectional study conducted from January to March, 2012. Ethical permission was obtained from the Enugu State Educational Board. Informed consent was obtained from parent or guardian of school children one week before sample collection. The community primary school in the three communities was sampled. School children were randomly selected and only those 4-15years were recruited into the study. School children within this age group who declined after selection were excluded.

### Sample collection and analysis

Urine samples were collected from school children in well labelled, clean, wide mouthed containers with screw caps between 10:00am and 2:00pm. The samples were immediately moved to the laboratory of the Department of Zoology and Environmental Biology, University of Nigeria. The presence of blood and protein in urine were determined using reagent strips (Medi-test Combi-9, manufactured by Machery-Hagel, Duren, Germany). Urine samples were gently agitated and 10ml drawn out using disposable syringe. The 10ml urine samples were concentrated by sedimentation for 30mins and supernatant carefully drawn without disturbing the sediment. The entire sediment was examined under the microscope and egg counts were recorded. Cases of schistosomiasis were defined as children with at least one *S. haematobium* egg on microscopic examination of urine. Intensities of *S. haematobium* infection was categorized as light (<50eggs/10ml urine) and heavy (≥50 eggs/ 10ml urine) infections (8). Infected children were given 40 mg/kg praziquantel.

### Snail sampling

Water contact sites in these communities were identified and sampled for snails for 12months using fine mesh kitchen strainers. Where necessary, snails were hand-picked and precautions taken by using a pair of gloves. The snail sampling was done by two collectors for 30minutes and the number of snails collected were counted and recorded. The first site is Nike Lake, which approximately 0.5km from the community school and residential areas in Amorji. The Lake is approximately 2km from Ibagwa and 1.5km from Amokpo. The second site is Iheugba, a stream that dries up during dry season. This stream is approximately 10km away from Amorji, 1km way from Ibagwa and 7km away from Amokpo. Iyi-oku is a river, with a distance of approximately 0.2km from Ibagwa, 1.5km from Amorji and 4km from Amokpo. The fourth site is Ava, a river that flows very fast in rainy season and cuts across Amorji and Amokpo, and is 3km from Ibagwa. After collection, snails were taken to the laboratory where they were identified using the field guide to West African fresh water snails prepared by the Danish Bilharziasis Laboratory, Denmark (9). Schistosome intermediate host identified were screened for cercariae using the shedding and crushing methods. Using the shedding method, snails were placed individually into flat bottom glass vials containing clean water and exposed to light for four hours. Snails that did not shed cercariae were pressed and crushed between two microscope slides. Cercariae were identified as described by (10, 11).

### Data analysis

Version 17.0 of the SPSS for windows software was used to analyse data. Age of school children was classed into three: 4-7years, 8-11years and 12-15years. Significant difference in the prevalence of infection by age and gender were determined using chi-square (X^2^) test. Significant differences in mean egg count between age groups and gender were determined using one-way analysis of variance (ANOVA) and t-test respectively. Relationship between presence and intensity of *S. haematobium* eggs with micro-haematuria was determined using Pearson Correlation. All analysis was done at *P* < 0.05.

## Results

### Parasitological findings

A total of 323 (9.02 ± 2.86 years) school children were examined, comprising 48.61% females (n= 157, 8.89 ± 2.71years) and 51.39% males (n = 166%, 9.14 ± 3.00 years). Children 4-7years of age were 16 (4.95%) while those 8-11 and 12-15years were 156 (48.30%) and 151 (46.75%) respectively.

Overall prevalence of urinary schistosomiasis was 4.64% (n = 15). The prevalence of the infection was insignificantly higher (Χ^2^=0.024, P =1.0) in males (4.8%, n = 8) than in females (4.5%, n = 7). *Schistosoma haematobium* infection was not detected in the age group 4-7years while the prevalence of the infection in those 8-11 and 12-15years was 4.5% (n = 7) and 5.3% (n = 8) respectively. The difference in prevalence in age groups was also not significant (X^2^=0.934, *P* = 0.627) (Table 1).

**Table 1:** Prevalence and intensity of *S. haematobium* infection among school children stratified by gender and age

	NO. examined	Positivity n (%)	Intensity n (%)		
Gender			Mean (SD) egg/10ml urine	Heavy infection	light infection
**Males**	166	8(4.8)	1.22(0.60)	2 (1.2)	6 (3.6)
**Females**	157	7(4.5)	1.06 (0.56)	1 (0.6)	6 (3.8)
**Age group(yr)**					
**4-7**	16	0(0)	0 (0)	0 (0)	0(0)
**8-11**	156	7(4.5)	1.39 (0.71)	2 (1.3)	5 (3.2)
**12-15**	151	8(5.3)	0.99 (0.49)	1 (0.7)	7 (4.6)
**Total**	323	15(4.6)	1.14 (0.41)	3 (0.9)	12 (3.7)

The mean ova count of school children was 1.14 ± 0.41 eggs/10ml urine. Although the mean ova count of males (1.22 ± 0.60 eggs/10ml urine) was more than that of the females (1.06 ± 0.56 eggs/10ml urine), there was no significant difference (*P* = 0.86) in their mean ova count. Children 8-11yrs had higher mean ova count than those 12-15yrs. However, no difference in mean ova count was observed statistically (*P* = 0.73). The proportion of school children with light infections was 3.7% while only 0.9% of the school children were heavily infected. Females (3.8%) had light infections than males (3.6%) while heavy infections were more prevalent in males (1.2%) than in females (0.6%). In the age groups, those 8-11 years had more heavy infection than those 12-15years while the reverse was observed with light infections (Table 1).

The prevalence of micro-haematuria (6.2%, n = 20) was higher than the prevalence of *S. haematobium* infection determined by microscopy. The prevalence of micro-haematuria in females and males were 6.4% (n = 10) and 6.0% (n = 10). Micro-haematuria was not detected in urine of children 4-7years. The prevalence of micro-haematuria in age group 8-11 and 12-15years was 8.3% (n = 13) and 4.6 (n = 7), respectively. Micro-haematuria correlated significantly with both the prevalence (r = 0.615, *P*<0.0001) and intensity (r = 0.5, *P*<0.0001) of *S. haematobium* eggs.

### Snail sampling

**Fig. 1:**
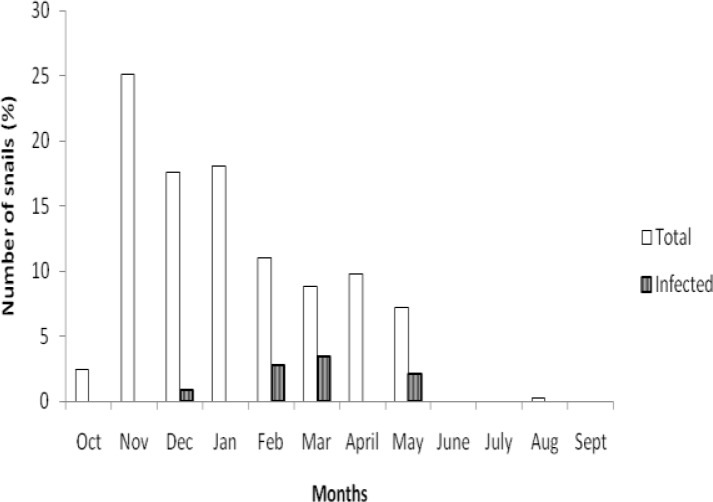
Monthly variation in the number of *B. globosus* collected and infected with *S. haematobium* in Nike Lake area of Enugu State

In Nike Lake, *Bulinus globosus, B. senegalensis, Lymnaea natalensis* and *Biomphalaria pfeifferi* were found. Snail composition in Ava River was *L. natalensis* and *B. globosus*. In Iyi-oku, *Lanistes* spp was the only snail found while in Iheugba stream no snail was found. A total of 677 *B. globosus* and 180 *B. senegalensis* were collected from the area. Only *B. globosus* shed *Schistosoma* cercariae, with snail infection rate of 0.73%. Infected snails were found in December, February, March and May (Fig. 1).

## Discussion

The prevalence rate of 4.6% confirms that *S. haematobium* infection is also prevalent in this urban part of Enugu State although the prevalence and intensity is lower than previously reported in rural areas of the State. However, similar low prevalence has been reported in some primary schools in the urban city of Ibadan, south-western, Nigeria (12).

The insignificant higher prevalence and intensity of infection in males than females still depict the male bias that exists in schistosome infection despite the low prevalence of infection. This may be attributed to higher cercarial exposure in males as opposed to any kind of sex differences in biological mechanism of resistance or egg output (13). Similar results of no significant difference in the prevalence of *S. haematobium* infection between males and females have been reported in neighbouring Anambra State by (14, 15).The zero prevalence found in school-children 4-7yr could be attributed to the fact that this age group only accompany the older ones to the open water bodies but are not actively involved in activities that take place at the water contact sites because of their age and fear of drowning.

The use of blood detected by reagent strip has been considered an alternative in the diagnosis of *S. haematobium* infection. As observed in the present study, blood in urine has been shown to have significant positive correlation with presence and intensity of the infection because the quantity of blood passed out increases with intensity of infection (16, 17). The higher prevalence of *S. haematobium* infection using micro-haematuria as diagnostic marker suggests the daily variation of *S. haematobium* egg excretion in infected individuals (18).

*Bulinus truncatus* was implicated as intermediate host of *S. haematobium* infection in Amangunze, Enugu State (5) but in the present study no *B. truncatus* was collected while the *S. haematobium* shedding snail host collected is *B. globosus*. The *B. senegalensis* collected did not shed *Schistosoma* cercariae. However, in Senegal River Basin, *B. senegalensis* has been identified as the intermediate host of urinary schistosomiasis (19). Consistent with the result from Amagunze village in Enugu State (5), the *Biom. pfeifferi* collected did not shed schistosome cercariae. The shedding of *S. haematobium* cercariae from *Bulinus globosus* was mostly in the dry season, which coincides with when wells dry up. It has been observed that the prevalence of digenean cercariae is usually higher in dry season than rainy season. Possibly, due to low volume of water in conjunction with increased density of snail host and intensified use of habitat by definitive host (20).

## Conclusion

Urinary schistosomiasis is prevalent in Nike Lake area of Enugu State. Pipe-borne water source should be made functional especially during dry season. Furthermore, urban areas as this should not be neglected in MDA control programmes. There is also need for community health education on this neglected tropical disease in the area as most people have no knowledge of the disease, the intermediate host and the causative organism.
